# Frost Damage Index: The Antipode of Growing Degree Days

**DOI:** 10.34133/plantphenomics.0104

**Published:** 2023-10-04

**Authors:** Flavian Tschurr, Norbert Kirchgessner, Andreas Hund, Lukas Kronenberg, Jonas Anderegg, Achim Walter, Lukas Roth

**Affiliations:** ^1^ Department of Environmental System Sciences, Institute of Agricultural Sciences, ETH Zürich, Zürich, Switzerland.; ^2^ Crop Genetics, John Innes Centre, Norwich, UK.; ^3^ Department of Environmental System Sciences, Institute of Integrative Biology, ETH Zürich, Zürich, Switzerland.

## Abstract

Abiotic stresses such as heat and frost limit plant growth and productivity. Image-based field phenotyping methods allow quantifying not only plant growth but also plant senescence. Winter crops show senescence caused by cold spells, visible as declines in leaf area. We accurately quantified such declines by monitoring changes in canopy cover based on time-resolved high-resolution imagery in the field. Thirty-six winter wheat genotypes were measured in multiple years. A concept termed “frost damage index” (FDI) was developed that, in analogy to growing degree days, summarizes frost events in a cumulative way. The measured sensitivity of genotypes to the FDI correlated with visual scorings commonly used in breeding to assess winter hardiness. The FDI concept could be adapted to other factors such as drought or heat stress. While commonly not considered in plant growth modeling, integrating such degradation processes may be key to improving the prediction of plant performance for future climate scenarios.

## Introduction

The ongoing climate change poses major challenges for food production. With increasing variability in climate extremes, adapted crops and agronomic practices to such stressors are urgently needed [1]. Frost stress is an important but little studied weather extreme, causing essential damage to crops such as winter wheat [2–5]. Most of the daily calorie intake is covered by a few arable crops including wheat. Identifying and minimizing the risks in the cultivation of these crops are therefore urgently needed [6]. The predicted decline in global wheat production and quality (i.e., protein content and composition) due to global climate change further underpins these findings [7,8]. Corresponding mitigation measures include either diversification of cropping systems or breeding for crops with improved performance under stress [9].

**Fig. 1.**
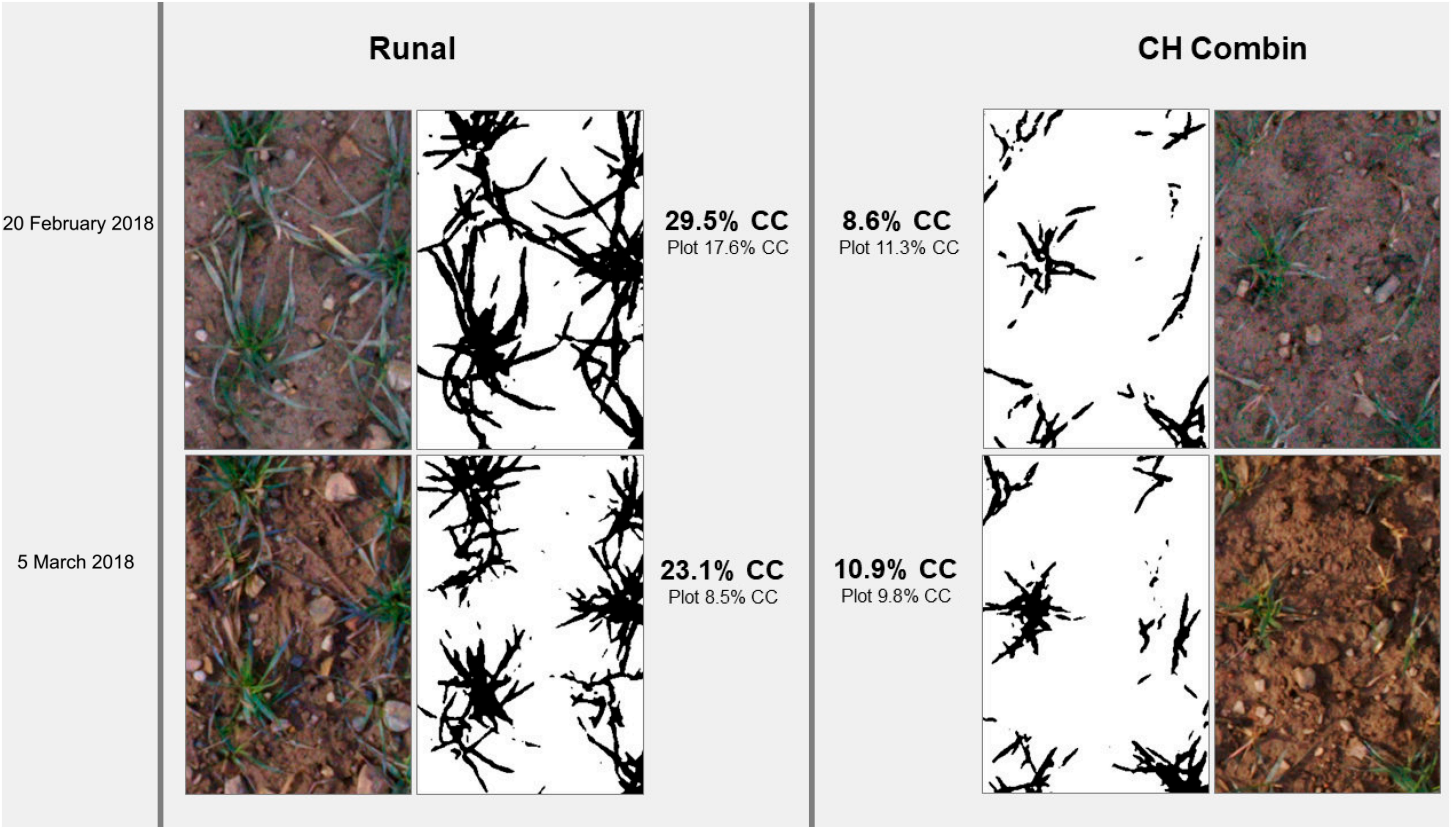
HTFP-based RGB image (color), plant-soil segmentation (black/white), and extracted CC per area, and plot (%) for 2 wheat varieties, Runal (susceptible to freezing stress) and CH Combin (more tolerant to freezing stress), before (20 February 2018) and after (5 March 2018) a cold spell.

**Fig. 2.**
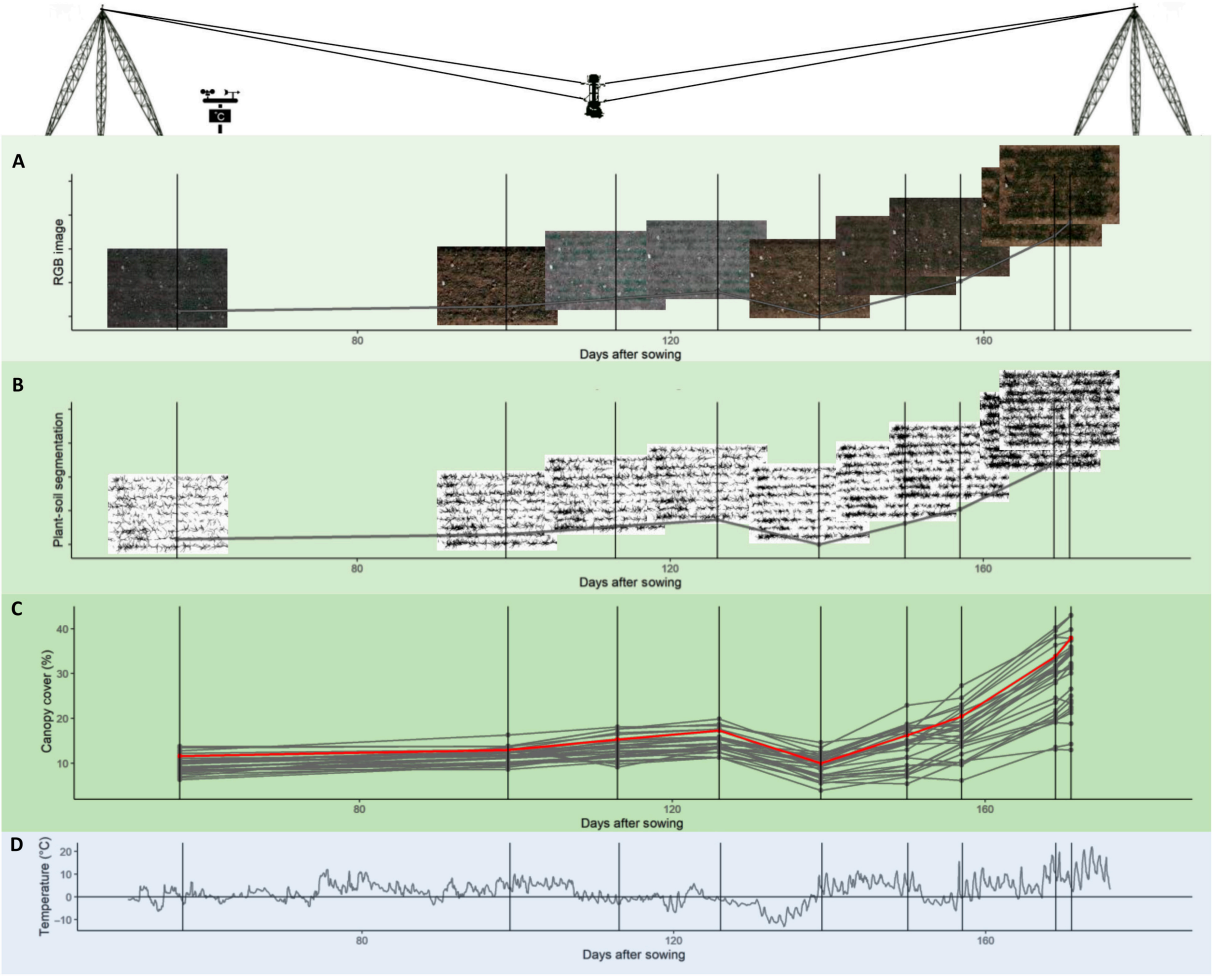
Overview of the data collection and processing workflow for CC, measured with the FIP using the example of 2018 and the variety Ludwig. The FIP enabled to take RGB images repeatedly throughout the growing season (A). RGB images were then segmented in plant and nonplant pixels (B). From these images, the CC per specific area was extracted (C). In addition to RGB images, temperature data were collected from a nearby weather station (D). The RGB and segmented images (A and B) and red line in (C) depict the variety Ludwig, and gray lines in (C) depict all other examined varieties.

In the search for adapted and resilient crops, modern phenotyping methods have shown great potential. Consequently, phenotyping the dynamic development of crops has become a relevant research field. In particular, the use of cameras in the context of plant phenotyping has been explored, and substantial progress has been made in feature extraction and subsequent data analysis ([10–13]). Modern plant phenotyping methods were first established under controlled conditions in climate chambers [14–16] and later also in the field [17–19]. A range of methods and sensors has evolved, and image-based high-throughput field phenotyping (HTFP) is widely used in research, especially in wheat [20,21]. Various growth-related and yield-relevant traits, such as canopy height [22], canopy cover (CC) [23] (Fig. 1), greenness [24,25], or ear density [26], can be estimated from images. Contemporary deep learning image analysis techniques facilitate the extraction of such traits, i.e., by classifying soil and plant areas in images to derive CC. Images can be taken by cameras mounted on diverse carrier systems such as tractors, phenomobiles, unmanned aerial vehicles (UAV), or stationary HTFP platforms. An HTFP platform is operated in the group of Crop Science at ETH Zürich, known as the field phenotyping platform (FIP) [27], a rigged sensor system (Fig. 2). The FIP provides very high-resolution red–green–blue (RGB) images captured from a short distance to the crop (on average of 2.5 m above the canopy, <0.3-mm ground sampling distance). Unlike other phenotyping platforms such as UAVs or ground-based robots, the image acquisition in the FIP is, for the most part, not limited by technical constraints such as rotor downwash or battery capacity or by environmental constraints such as unfavorable soil and weather conditions [28]. Hence, the FIP allows gathering unique datasets with high temporal and spatial resolution, supporting the extraction of traits down to the scale of individual plant organs. Such detailed, spatial information is often not captured using other sensors (e.g., point sensors). Hence, changes in the canopy are not directly absolutely quantifiable [4].

Autumn-sown winter wheat is frequently exposed to freezing temperatures during its early development in fall, winter, and early spring [29,30]. Winter wheat has a certain winter hardiness, but too low temperatures can lead to freezing damage (Fig. 1) [4]. Frost events are predicted to occur also in future climates and particularly in early spring season [3,31]. Severe damage during and especially at the end of winter can hinder or slow down the development of the plant, decreasing its yield potential [2]. Assumed reasons for such a reduced yield potential are, among others, reduced tillering [32], slower nutrient uptake [33], or reduced competitiveness against weeds [34] due to a less pronounced early vigor.

The resilience toward frost stress is known to be genotype specific [35] (Figs. 1 and 2). However, most approaches to modeling frost damage lack a genotype-specific analysis, presumably due to too coarse spatial scales (e.g., [4,5]). Frost damage during the vegetative growth stages manifests itself in the form of dead, brown leaves. Quantification for breeding purposes is often done with a visual scoring by eye to grade the severity of the damage [36]. However, such laborious and subjective visual scorings are limited in their precision and in their potential to reveal a genotype’s dynamic response to cold stress and, in particular, its recovery from it. Alternatively, time series of CC, defined as the proportion of visible projected healthy (i.e., green) leaf area per ground area, can be used to determine damage. The high spatial and temporal resolution of time series collected with the FIP enables to detect even small changes in the CC of plants on sowing row level precisely. Consequently, reductions in the CC after cold spells can be observed.

**Fig. 3.**
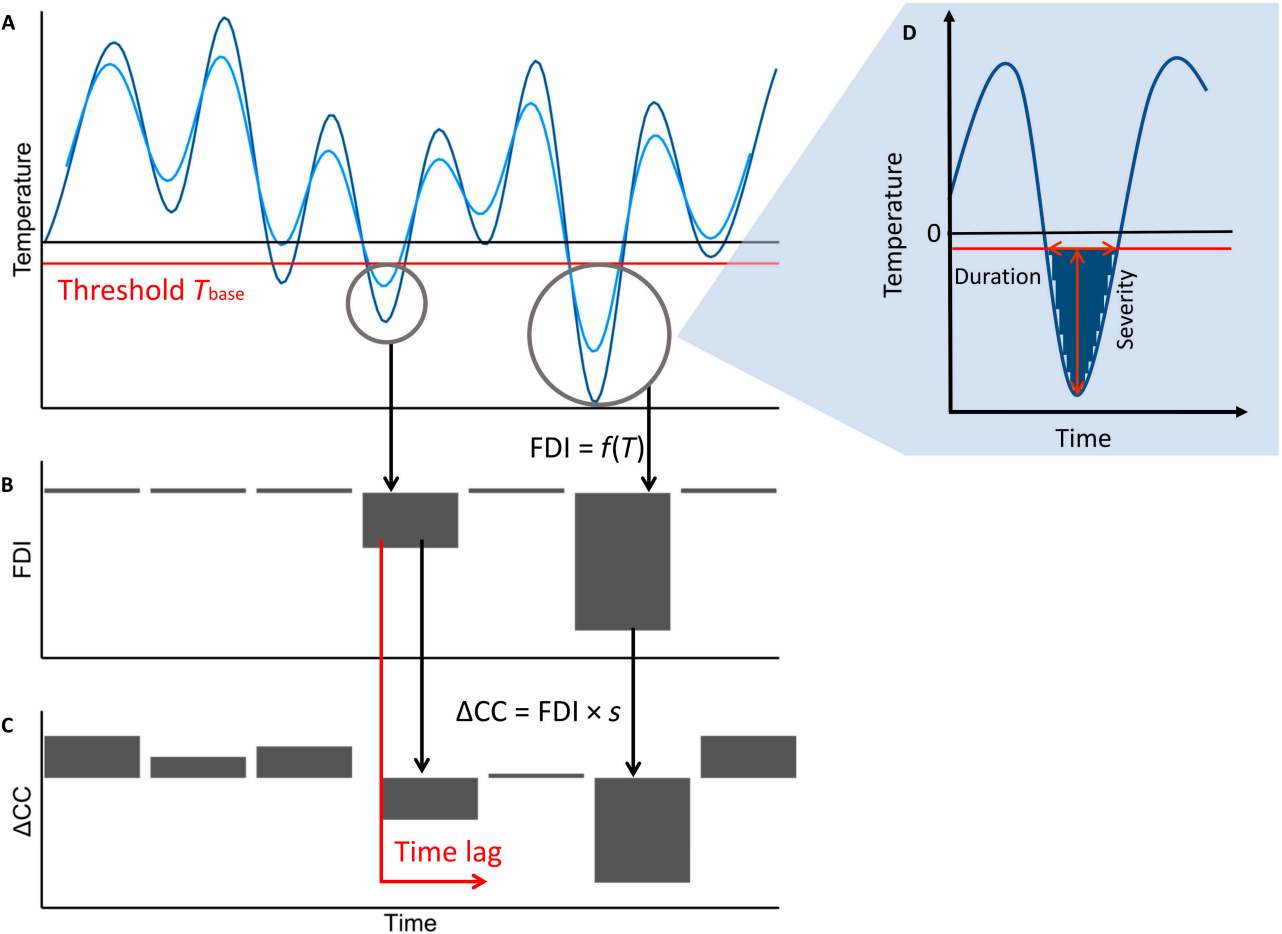
The concept of the FDI (B) based on air-temperature measurements (A) and its relation to CC decline (C). The FDI is defined as the sum of temperatures below a certain threshold (A, red line), therefore summarizing the duration and severity of an event (D), with no frost stress leading to zero values and stress leading to negative values (B). Measured CC changes (ΔCC) are positive when no stress occurs and negative when stress occurs (C). Relating the FDI to declining CC requires 2 additional parameters, the time lag between frost events and visible damage (B and C, red arrow) and the sensitivity to the FDI (B and C, black arrows). The time lag allows to account for a delayed appearance of CC decline after a frost event, e.g., due to delayed biotic and/or abiotic processes. Temperature courses may be smoothed before deriving the FDI [light blue versus dark blue lines in (A)], and smoothing reduces the leverage of extreme frost events.

Widely used for describing growing processes are growing degree days (GDDs) [37]. GDDs consider the temperature sum above a certain (crop-specific) threshold temperature. Using this thermal time instead of the ordinal time is supposed to lead to a linearized growth process. In this line, we develop the concept of “frost damage index” (FDI), which incorporates the severity and duration of frost events that determine the (potentially irreversible) damage to the plant [38,39]. For frost events, the severity (defined as the amplitude of the frost event) indicates that frost damage will occur, as plants show a certain adaptation to temperatures before cells are irreparably damaged [40]. Including the duration of an event below this temperature, via the temperature sum, allows to quantify the occurring damage (Fig. 3D). The extent of freezing damage was shown to be influenced by the severity of the exposed temperature stress [4]. Smoothing of the measured temperature courses may be used to reduce the detected number of frost events and thus to improve the relationship between FDI and the observed damage [Fig. 3A, dark (raw) versus light blue (smoothed) trajectory]. In addition, considering a lag time between stress event and visual damage allows to incorporate biological processes of degradation, which are known to take time and may be delayed by low temperatures themselves (Fig. 3C). The FDI itself is consequently dependent on one parameter, the base temperature (*T*_base_) that determines the temperature below which damage can occur,

$$\mathrm{FDI}\left(t_{n};\;T_{\mathrm{base}}\right)=\sum_{t=t_{n-1}}^{t_{n}}\text{min}\left(T_{t}-T_{\mathrm{base}},0\right)$$ (1)

where *T_t_* are hourly temperature values between 2 trait measurement time points *t*_*n* − 1_ and *t_n_* (Fig. 3D). *T_t_* − *T*_base_ represents the severity of the event, while cumulating these stress hour amplitudes integrates over the entire duration of the event. The FDI may then be multiplied by a (potentially genotype specific) parameter *s*, which is a scaling/sensitivity factor, allowing to relate the index value to the corresponding measurement (i.e., change in ΔCC),

$$\Delta \mathrm{CC}\left(t_{n}\right)=\mathrm{FDI}\left(t_{n};T_{\mathrm{base}}\right)\cdot s$$ (2)

On the basis of the concept of the FDI, this study aims to identify the base temperature, optimal temperature smoothing, and lag time that allows the quantification of frost damage based on the relationship between observed temperatures and changes in CC.

## Materials and Methods

Field experiments were conducted at the ETH research station for plant sciences Lindau-Eschikon, Switzerland (47.449°N, 8.682°E, 520 m a.s.l.). Measurements were performed with the FIP [21] in the years 2018, 2019, and 2021. The study was examined on a set of 36 Swiss elite winter wheat varieties. Wheat varieties were grown in plots with a size of 1.125 × 1.75 m in 9 rows with 12.5-cm distance between each row. Sowing was done on 17 October 2017, 17 October 2018, and 17 October 2020, respectively, with a sowing density of 400 kernels/m^2^. The temperature data were derived in hourly resolution from a nearby weather station located at approximately 50-m distance from the field trial.

An RGB 21 MP full frame digital single-lens reflex (DSLR) camera (EOS 5D Mark II, 35 mm lens; Canon Inc., Tokyo, Japan) attached to the FIP was used for the measurements, delivering a ground sampling distance of 0.3 mm per pixel. The measurements were conducted according to weather conditions (e.g., no heavy rain or snow cover). This resulted in one measurement per week on average in the early season (Fig. 2). RGB images were segmented pixel-wise into a plant and a soil fraction using a deep convolutional neural network as described previously, with an overall accuracy of above 0.95 [41].

To align time series of plot images, planar homography estimation was applied. The SIFT (scale-invariant feature transform) [42] and ORB (oriented FAST and rotated BRIEF) [43] algorithms served as feature detection methods for subsequent image pairs in the time series. The detected matching features were then processed with the RANSAC (random sample consensus) [44] algorithm to find the homography matrix between image pairs, which provides a certain robustness against outliers. Feature detection and homograph estimation were performed in Python using OpenCV.

To enable identification of individual plant rows on previously aligned images, the segmented image (showing plant and soil pixels) was further rectified: First, a brute-force approach was applied to rotate the image from −1.5° to 1.5° in steps of 0.2°. For each iteration, the sum of plant pixels per image column was calculated. These sums were then filtered using a mean sliding window of size 100, and the difference between the maximum and minimum value was extracted. The rotation where this difference was maximized was chosen as the optimal rotation.

**Fig. 4.**
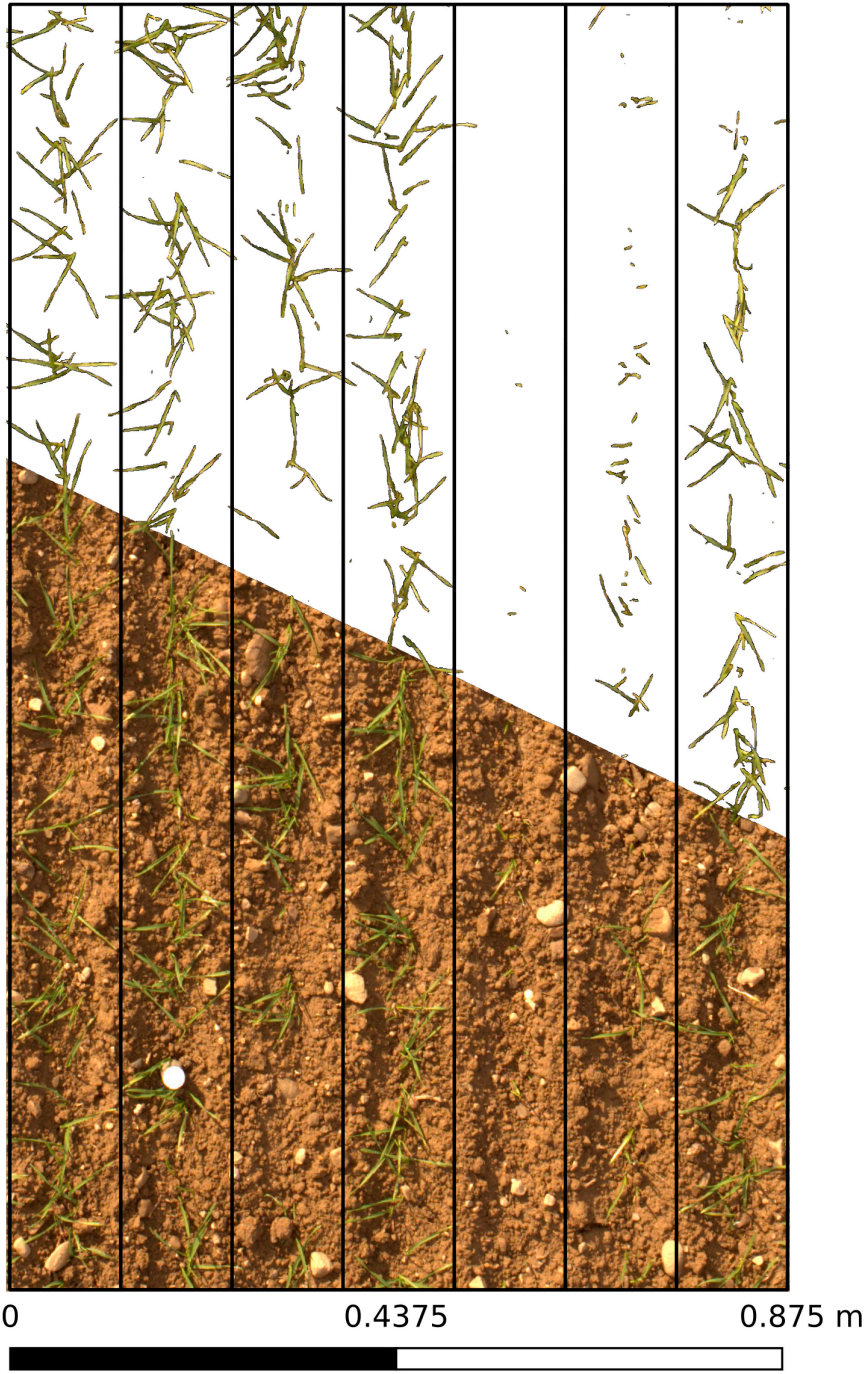
Rotated and scaled original image (bottom) and segmented plant pixels (top) with identified sowing rows (black rectangles) and plot shape (outer bound).

After rotating the image accordingly and recalculating the column sums, a 9-fold sinus pattern was fitted to the column sums, representing the 9 sowing rows of each plot on the field. From these 9 sowing rows, the inner 7 sowing rows (black rectangles in Fig. 4) were used for further processing. CC was then calculated as plant pixel ratio per sowing row. All processing was performed in Python using scipy [45].

On the basis of the CC measurements, canopy growth can be described as the difference in CC between 2 measurement time points *t_n_*,

$$\Delta \mathrm{CC}\left(t_{n}\right)=\mathrm{CC}\left(t_{n}\right)-\mathrm{CC}\left(t_{n-1}\right)$$ (3)

where *n* is the *n*th measurement time point of *N* measurements with *n* = (2, …, *N*). Positive ΔCC values represent an increase in the CC and hence growth. Negative values of ΔCC show a reduction of the CC and, hence, damage to the plant. Given that the measurement period was restricted to early spring, other factors than frost damage (e.g., heat and pest stress and natural plant senescence) can be excluded with high probability. Consequently, the measured reduction in CC was most likely caused by frost events. These negative ΔCC values were considered for the subsequent fitting steps. In addition to the CC measurements, a visual scoring of frost damage was done using the Fieldbook-App [46] according to the frost tolerance scheme of [36]. Canopies were given a grade between 1 (absolute frost tolerance) that represents no visible frost damage at all and 9 that represents a complete dead plant.

**Fig. 5.**
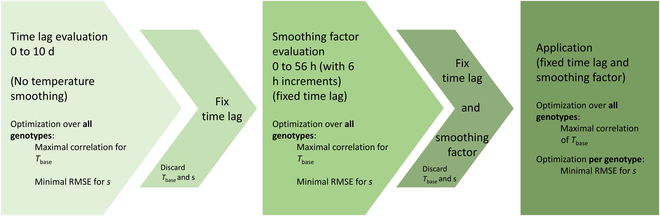
Three steps of the evaluation and parameter estimation process: the first 2 on row-based levels using data from all genotypes and the third step on a genotype-specific level.

According to visual scorings and temperature courses, frost events were present in all 3 years, but timely image data could be acquired in 2018 and 2021 only. In 2019, the first image acquisition following the major frost event was—due to unfavorable weather conditions—carried out only after a few weeks (Fig. S1). Nevertheless, visual scorings were available for this year and for 2018. Consequently, parameters constituting the FDI were optimized on the basis of 2018 and 2021 data but were validated in all 3 years. For the parameter estimation steps (Fig. 5), row-based values (7 rows per plot, 1 to 9 plots per genotype, and 36 genotypes within the 3 years) were used.

When quantifying frost damage, in addition to *T*_base_ and *s*, 2 additional general factors have to be taken into account: (a) The visible damage to the plants may have a certain time lag to the cold spell that occurred, and (b) the severity and length of a cold spell influence the visible damage to the plants, which can be accounted for using a temperature course smoothing factor.

All parameters were estimated using a step-wise evaluation (Fig. 5). First, the optimal crop-specific time lag was evaluated using all available genotypes. For this purpose, all ΔCC measurements were interpolated to daily values to reduce bias according to varying measurement intervals. Then, the optimal time lag was determined by evaluating time lags between 0 and 10 d and selecting the value that results in the highest prediction accuracy (Pearson’s correlation coefficient) and lowest root mean square error (RMSE). In a second step, with a fixed time lag set to the optimal value from the previous step (by adding the optimal time lag to the temperature trajectory), a smoothing factor between 0 and 56 h (6 h increments) was applied to the temperature course. Again, its optimal value was selected on the basis of the prediction accuracy and RMSE. The smoothing was applied via a 2-sided moving average function, which was fixed and applied to the temperature trajectory for the next steps in the analysis. For the 2 steps described above, *T* _base_ and *s* had to be estimated as well and were optimized on a global level (genotype-unspecific) but discarded afterward. After optimizing the time lag and smoothing factor and fixing them, *T* _base_ was re-estimated on a global level, and *s* was re-estimated on a global and genotype-specific level.

For both the initial estimation and the re-estimation after fixing the time lag and smoothing factor, in a first iteration, the parameters *T*_base_ was optimized using Pearson’s correlation (cor) maximization,

$${\hat{T}}_{\text{base}}\underset{\text{argmax}}{=}\mathrm{cor}\left(\Delta CC\left(t_{n}\right),\;\mathrm{FDI}\left(t_{n};\;T_{\mathrm{base}}\right)\right)$$ (4)

Optimizing Pearson’s correlations instead of errors were preferred as this prevents the solution from predicting the global mean or zero.

In a second iteration, the parameter *s* was optimized using RMSE minimization,

$$\hat{s}\underset{\text{argmin}}{=}\sqrt{\frac{1}{N}\;\sum_{n}^{N}{\left(\Delta \mathrm{CC}\left(t_{n}\right)-\mathrm{FDI}\left(t_{n}\;;\;{\hat{T}}_{\text{base}}\right)\times s\right)}^{2}}$$ (5)

Optimizing for low RMSE was chosen as it relates to the target of predicting the genotype-specific sensitivity to winter kill with the lowest possible error.

All optimization was done in R [47] using the optim and BBoptim [48] functions of the stats package using the limited memory algorithm for bound constrained optimization [49].

## Results

The temperature courses for the 3 different years varied, and cold spells of different amplitudes occurred. In the following, we focus on the first 150 days after sowing (DAS) for each year (Fig. S1), which represents the winter period (Fig. S1). The year 2018 was characterized by a relatively mild winter with a very severe cold spell in the late winter around 140 DAS, which corresponds to the time point where negative ΔCC values were measured. The period of 2019 showed lower temperatures on average, with less severe cold spells around 120 DAS. The third year, 2021 was comparable to 2019 in terms of mean temperature but showed more severe cold spells around 120 DAS. The measured decrease in CC (ΔCC) on a plot level was moderately to strongly correlated to manual frost ratings after a cold spell for 2018 (*r* = 0.62, FIP measurement at 140 DAS) and 2019 (*r* = 0.49, FIP measurement at 121 DAS) (Fig. S2 and Table S1). Across the 3 observed years, 2019 showed the highest CC values and the fastest growth in CC. In 2018 and 2021, a moderate growth during the winter phase with a strong increase in CC at the end of the analyzed period was observed.

In the first parameter estimation step, the evaluation of the time lag between cold spell and visible damage in CC resolved in an optimal lag time of 3 d. The lag of 3 d maximized prediction accuracy (Pearson’s correlation coefficient) and minimized RMSE (Fig. S3). Therefore, 3 d of lag was used for the further processing steps.

In the second parameter estimation step, the smoothing factor was evaluated using again pooled (genotype-unspecific) values. The results indicated a positive effect of smoothing (using a moving average) on the prediction accuracy, with a maximum at a sampling window size of 12 h (Fig. S4). Consistent with this, the RMSE decreased when a wider sampling window was used, with a minimum at 24 h. Optimizing the smoothing factor increased the prediction accuracy from 0.75 up to 0.83 and, hence, decreased the RMSE as well. However, the effect on the prediction accuracy and RMSE was less pronounced as of the time lag. Consequently, a smoothing period of 18 h was used for further processing steps, which represents a compromise between optimal prediction accuracy and RMSE.

In a third step, time lag and smoothing factor were fixed to the previously optimized values, and *T*_base_ was optimized using row-based values of all available genotypes. An optimal value of −9 °C was found (Fig. S5).

**Fig. 6.**
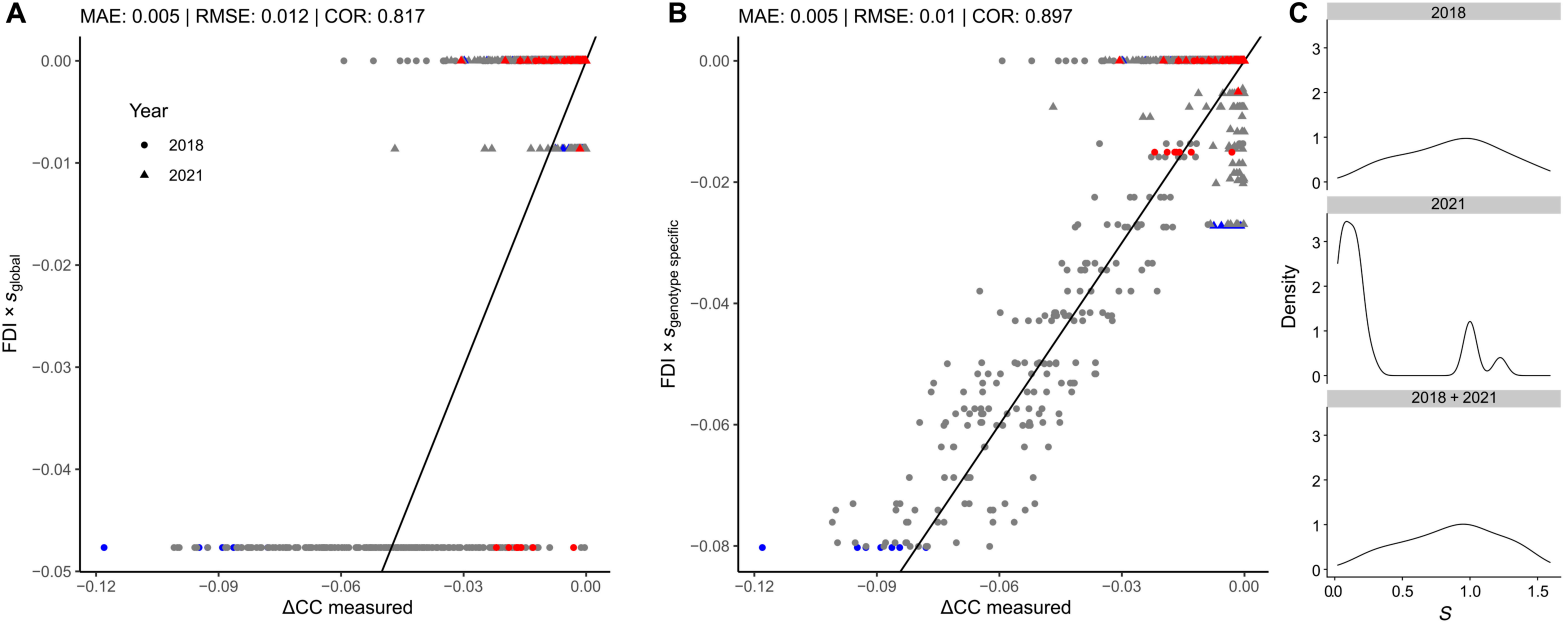
Predicted frost damage based on the FDI plotted against CC decline (ΔCC measured per sowing row). A time lag of 3 d and temperature course smoothing with an 18-h moving average were applied. The solid line shows the 1:1 relation, red dots represent the marked variety CH Combin, and blue dots indicate the variety Runal (see Fig. 1). The different years are depicted by the shape. The optimization was once performed with fixed *T*_base_ to −9 °C and a global parameter *s* (A), and once with fixed *T*_base_ to −9 °C and a genotype-specific parameter *s* (B). The distribution of genotype-specific estimations for *s* for single years (2018 and 2021) and the multiyear combination are shown in (C). MAE, mean absolute error.

Finally, the sensitivity factor *s* was optimized for single genotypes and years as well as for 2018 and 2021 combined (Fig. 6C). While the distribution of estimated sensitivities for 2018 and the combined dataset 2018/2021 were comparable, the distribution for 2021 was very uneven, and most values were close to zero, indicating that data from 2018 contributed more to reasonable sensitivity estimations than data from 2021. Validation of the final FDI was done using visual ratings, considered as “gold standard”. These visual ratings have been correlated with ΔCC measurements at a specific date and with the FDI (the sensitivity factor *s*), thus providing indications of the validity of our approach on these 2 processing steps (Table S1).

Predicting ΔCC using a genotype-specific *s* yielded a prediction accuracy of 0.897 with an RMSE of 0.01 and a mean absolute error of 0.005 (Fig. 6B). When not considering the sensitivity factor *s* as genotype specific, lower prediction accuracy and higher RMSE were found (Fig. 6A). Low FDI values were generally related to severe frost damage; however, the FDI tended to underestimate smaller frost damage (Fig. 6). When relating the sensitivity factor *s* to manual winter kill ratings in 2018, a Spearman’s rank correlation coefficient of 0.62 was found. In addition, genotype-specific *s* values for 2018 and 2021 were related to 2019 visual scorings with a Spearman’s rank correlation coefficient of 0.48. These estimations for *s* based on 2018 and 2021 data also correlated with negative ΔCC measurements made in 2019 at 121 DAS with a coefficient of 0.52 (Table S1).

## Discussion

The FDI allowed an accurate prediction of frost damage during the winter period based on temperature measurements. Prerequisite for the prediction was an optimization of the length of the lag phase, an optimal smoothing factor, a critical threshold temperature *T*_base_, and a genotype-specific sensitivity to the FDI (*s*). Predictions were more accurate for severe damage events compared to less severe damage events (Fig. 6). Causes for the observed small and FDI-insensitive CC decrease events may reach beyond temperature as the only stressor: Plant diseases or natural plant senescence during the winter period may cause CC decline as well. In addition, differentiating between FDIs for severe stress and weak stress events caused by temperature, and determining separate sensitivity factors for both FDIs may further improve the predictions. However, testing this hypothesis would require large datasets and, even more importantly, a more balanced distribution of sever and weak stress events across multiple years.

The prediction accuracy of the FDI regarding the measured damage increased considerably when a lag phase was taken into account. The optimal period for a lag phase appeared to be 3 d. The hill-shaped performance curve (Fig. S3) with an optimum at a lag of 3 d indicated that damage is also measurable before and after the optimum, as physical plant damage may appear as a normal distribution over time. Therefore, a perfect correlation is never achieved by setting a specific lag. In addition, the lag phase may be delayed or shortened in specific years depending on the occurrence of particularly high or low temperatures after freezing events.

Smoothing temperature courses increased the prediction accuracy, but the importance of smoothing was, compared to the effect of the time lag, low. The smoothing factor and the time lag do compete with each other to some extent. Our intention behind the smoothing factor was to explicitly state what is often implicitly done: Many studies that integrate temperature, e.g., using GDDs, work with daily mean temperatures. Such aggregation approaches imply a smoothing and reduction of leverage of extreme events but do not declare how this smoothing is exactly achieved. By using hourly temperature values and optimizing the smoothing parameter, we intended to become more transparent in this regard.

Despite the additional optimization of the lag and smoothing factors, our results revealed that −9 °C is a threshold temperature (*T*_base_) below which visible frost damage occurs. This result is in line with previous findings [35]. Furthermore, the sensitivity parameter *s* depends strongly on the data of a specific year. With many stress events and severe frost damage, 2018 showed a close-to normal distribution of the estimates of *s* (Fig. 6C). Thus, *s* is the most important driving variable contributing to genotype-specific frost damage, while the time lag, temperature smoothing, and *T*_base_ could be determined on a global level. Such global and therefore crop-specific parameters are also the base temperature of GDDs [50–52]. Therefore, the sensitivity of new genotypes to frost damage can be assessed using HTFP methods applied at a determined time point. This greatly simplifies the assessment of *s* for unseen genotypes. CC measurements are best carried out at well-chosen times, e.g., directly after a frost event when temperatures fell below −9 °C and again after the 3-d lag phase.

From a breeding point of view, the “application receipt” we provide enables an efficient and simple evaluation of frost-tolerant genotypes. The image-based, high-throughput manner of our method allows the screening of large populations and could easily be implemented on hand-held or vehicle-mounted platforms. Thus, the method would allow for QTL (Quantitative Trait Locus) screening and marker-assisted selection in larger breeding programs. Furthermore, the FDI could be used to improve crop models. In various growth models, damage effects are not taken into account, e.g., APSIM [53,54]. Simple indices such as the FDI could be incorporated without major changes into the original crop model to adjust growth according to specific stressors.

The introduced concept of the FDI index, which takes into account the length, severity, and lag of an abiotic stress, can also be used for other stressors. Along the same lines, indices could, e.g., be established that allow determination of genotype-specific heat stress damage [55–57]. For this purpose, the threshold temperature then would be a minimum temperature below which no damage occurs. Future studies will need to assess the validity of such a concept based on experimental data from the field.

In the long term, the ability to choose from an expanded range of environmental indices such as FDI could help accelerate genetic gain in breeding: It has been shown that the inclusion of genotype-specific responses to environmental indices such as maximum temperature, night temperature, and soil water status can improve genomic prediction models under strong genotype-by-environment interactions [58]. Integrating various abiotic stressors into a genomic selection framework to increase throughput and accuracy is widely suggested (e.g., [59–62]). Phenomic selection approaches based on measured genotype responses to covariates may offer similar advantages [63]. Recent developments in the use of factor-analytic linear-mixed models for genomic prediction [64] even allow the direct inclusion of indices of environmental covariates, forcing the model itself to find genotype-specific responses [65]. Considering that most of the indices investigated in such studies are related to heat stress, an extension of the available indices to the early growing period—and in particular to frost events—seems very promising.

The main value of this study is to establish, on the basis of field analyses, a method to relate the observed physiological damage of a plant to the severity of a causal stress. In this example, the relationship between growth and frost damage has been elucidated. This is a consistent further development of the concept of thermal time and will help to better characterize genotype-by-environment interactions. Such a concept can only be revealed with HTFP methods. Now, the door is open to application for basic science and cereal breeding alike.

## Data Availability

All code is available at https://gitlab.ethz.ch/ftschurr/fdi_example with example data. All data are available upon reasonable request.

## Supplementary Materials

Supplementary 1 Table S1. Spearman’s rank correlation coefficients between s and the visual scoring (VS) respectively of FIP measurements. Fig. S1. Overview of the 3 measurement years. Fig. S2. Visual ratings versus inverse ΔCC value for measurements in the year 2018 (days after sowing: 140) and 2019 (days after sowing: 212). Fig. S3. Prediction accuracy and RMSE over 0 to 10 d of lag time between FDI and daily interpolated ΔCC values. Fig. S4. Prediction accuracy and RMSE over 2 to 56 h of smoothing window of the temperature values. Fig. S5. Prediction accuracy using different *T*_base_ temperatures.

## References

[1] Seneviratne SI, Zhang X, Adnan M, Badi W, Dereczynski C, Di Luca A, Ghosh S, Iskandar I, Kossin J, Lewis S, et al. Weather and climate extreme events in a changing climate. In: Masson-Delmotte V, Zhai P, Pirani A, Connors SL, Péan C, Berger S, Caud N, Chen Y, Goldfarb L, Gomis MI, et al., editors. *Climate change 2021: The physical science basis. Contribution of working group I to the sixth assessment report of the Intergovernmental Panel on Climate Change*; Cambridge (UK) and New York (NY): Cambridge University Press; 2021. p. 1513–1766.

[2] Crimp SJ, Zheng B, Khimashia N, Gobbett DL, Chapman S, Howden M, Nicholls N, Crimp SJ, Zheng B, Khimashia N, et al. Recent changes in southern Australian frost occurrence: Implications for wheat production risk. Crop Pasture Sci. 2016;67(8):801–811.

[3] Xiao L, Liu L, Asseng S, Xia Y, Tang L, Liu B, Cao W, Zhu Y. Estimating spring frost and its impact on yield across winter wheat in China. Agric For Meteorol. 2018;260-261:154–164.

[4] Nuttall JG, Perry EM, Delahunty AJ, O’Leary GJ, Barlow KM, Wallace AJ. Frost response in wheat and early detection using proximal sensors. J Agron Crop Sci. 2019;205(2):220–234.

[5] Wang S, Chen J, Rao Y, Liu L, Wang W, Dong Q. Response of winter wheat to spring frost from a remote sensing perspective: Damage estimation and influential factors. ISPRS J Photogramm Remote Sens. 2020;168:221–235.

[6] Tilman D, Balzer C, Hill J, Befort BL. Global food demand and the sustainable intensification of agriculture. Proc Natl Acad Sci USA. 2011;108(50):20260–20264. 22106295 10.1073/pnas.1116437108 PMC3250154

[7] Asseng S, Ewert F, Martre P, Rötter RP, Lobell DB, Cammarano D, Kimball BA, Ottman MJ, Wall GW, White JW, et al. Rising temperatures reduce global wheat production. Nat Clim Chang. 2015;5(2):143–147.

[8] Asseng S, Martre P, Maiorano A, Rötter RP, O’Leary GJ, Fitzgerald GJ, Girousse C, Motzo R, Giunta F, Babar MA, et al. Climate change impact and adaptation for wheat protein. Glob Chang Biol. 2019;25:155–173. 30549200 10.1111/gcb.14481

[9] Reynolds M, Langridge P. Physiological breeding. Curr Opin Plant Biol. 2016;31:162–171. 27161822 10.1016/j.pbi.2016.04.005

[10] van Eeuwijk FA, Bustos-Korts D, Millet EJ, Boer MP, Kruijer W, Thompson A, Malosetti M, Iwata H, Quiroz R, Kuppe C, et al. Modelling strategies for assessing and increasing the effectiveness of new phenotyping techniques in plant breeding. Plant Sci. 2019;282:23–39. 31003609 10.1016/j.plantsci.2018.06.018

[11] Roth L, Piepho H-P, Hund A. Phenomics data processing: Extracting dose–response curve parameters from high-resolution temperature courses and repeated field-based wheat height measurements. In Silico Plants. 2022;4(1):diac007.

[12] Roth L, Rodríguez-Álvarez MX, van Eeuwijk F, Piepho HP, Hund A. Phenomics data processing: A plot-level model for repeated measurements to extract the timing of key stages and quantities at defined time points. Field Crop Res. 2021;274:108314.

[13] Pérez-Valencia DM, Xosé Rodríguez-Álvarez M, Boer MP, Kronenberg L, Hund A, Cabrera-Bosquet L, Millet EJ, Van Eeuwijk FA. A two-stage approach for the spatio-temporal analysis of high-throughput phenotyping data. Sci Rep. 2022;12(1):3177. 35210494 10.1038/s41598-022-06935-9 PMC8873425

[14] Rajendran K, Tester M, Roy SJ. Quantifying the three main components of salinity tolerance in cereals. Plant Cell Environ. 2009;32:237–249. 19054352 10.1111/j.1365-3040.2008.01916.x

[15] Kovalchuk N, Laga H, Cai J, Kumar P, Parent B, Lu Z, Miklavcic SJ, Haefele SM. Phenotyping of plants in competitive but controlled environments: A study of drought response in transgenic wheat. Funct Plant Biol. 2017;44(3):290–301. 32480564 10.1071/FP16202

[16] Kronenberg L, Yates S, Ghiasi S, Roth L, Friedli M, Ruckle ME, Werner RA, Tschurr F, Binggeli M, Buchmann N, et al. Rethinking temperature effects on leaf growth, gene expression and metabolism: Diel variation matters. Plant Cell Environ. 2021;44(7):2262–2276. 33230869 10.1111/pce.13958 PMC8359295

[17] White JW, Andrade-Sanchez P, Gore MA, Bronson KF, Coffelt TA, Conley MM, Feldmann KA, French AN, Heun JT, Hunsaker DJ, et al. Field-based phenomics for plant genetics research. Field Crop Res. 2012;133:101–112.

[18] Deery D, Jimenez-Berni J, Jones H, Sirault X, Furbank R. Proximal remote sensing buggies and potential applications for field-based phenotyping. Agronomy. 2014;4(3):349–379.

[19] Volpato L, Pinto F, González-Pérez L, Thompson IG, Borém A, Reynolds M, Gérard B, Molero G, Rodrigues FA. High throughput field phenotyping for plant height using UAV-based RGB imagery in wheat breeding lines: Feasibility and validation. Front Plant Sci. 2021;12:591587. 33664755 10.3389/fpls.2021.591587 PMC7921806

[20] Virlet N, Kasra S, Sadeghi-tehran P, Hawkesford MJ. Field Scanalyzer : An automated robotic fi eld phenotyping platform for detailed crop monitoring. Funct Plant Biol. 2017;44:143–153. 10.1071/FP16163 32480553

[21] Bai G, Ge Y, Hussain W, Baenziger PS, Graef G. A multi-sensor system for high throughput field phenotyping in soybean and wheat breeding. Comput Electron Agric. 2016;128:181–192.

[22] Kronenberg L, Yu K, Walter A, Hund A. Monitoring the dynamics of wheat stem elongation: Genotypes differ at critical stages. Euphytica. 2017;213:157.

[23] Roth L, Barendregt C, Bétrix CA, Hund A, Walter A. High-throughput field phenotyping of soybean: Spotting an ideotype. Remote Sens Environ. 2022;269: Article 112797.

[24] Wan L, Cen H, Zhu J, Zhang J, Zhu Y, Sun D, Du X, Zhai L, Weng H, Li Y, et al. Grain yield prediction of rice using multi-temporal UAV-based RGB and multispectral images and model transfer - a case study of small farmlands in the South of China. Agric For Meteorol. 2020;291:108096.

[25] Anderegg J, Tschurr F, Kirchgessner N, Treier S, Schmucki M, Streit B, Walter A. On-farm evaluation of UAV-based aerial imagery for season-long weed monitoring under contrasting management and pedoclimatic conditions in wheat. Comput Electron Agric. 2023;204: Article 107558.

[26] Fernandez-Gallego JA, Kefauver SC, Gutiérrez NA, Nieto-Taladriz MT, Araus JL. Wheat ear counting in-field conditions: High throughput and low-cost approach using RGB images. Plant Methods. 2018;14(1):1–12. 29568319 10.1186/s13007-018-0289-4 PMC5857137

[27] Kirchgessner N, Liebisch F, Yu K, Pfeifer J, Friedli M, Hund A, Walter A. The ETH field phenotyping platform FIP: A cable-suspended multi-sensor system. Funct Plant Biol. 2017;44:154–168. 10.1071/FP16165 32480554

[28] Roth L, Hund A, Aasen H. PhenoFly planning tool: Flight planning for high-resolution optical remote sensing with unmanned areal systems. Plant Methods. 2018;14(1):1–21. 30598692 10.1186/s13007-018-0376-6 PMC6302310

[29] Grieder C, Hund A, Walter A. Image based phenotyping during winter: A powerful tool to assess wheat genetic variation in growth response to temperature. Funct Plant Biol. 2015;42:387–396. 32480683 10.1071/FP14226

[30] Duan T, Chapman SC, Holland E, Rebetzke GJ, Guo Y, Zheng B. Dynamic quantification of canopy structure to characterize early plant vigour in wheat genotypes. J Exp Bot. 2016;67(15):4523–4534. 27312669 10.1093/jxb/erw227 PMC4973728

[31] Tschurr F, Feigenwinter I, Fischer AM, Kotlarski S. Climate scenarios and agricultural indices: A case study for Switzerland. Atmosphere. 2020;11(5):1–23.

[32] Valério IP, De Carvalho FIF, De Oliveira AC, Benin G, De Souza VQ, De Almeida Machado A, Bertan I, Busato CC, Da Silveira G, Fonseca DAR. Seeding density in wheat genotypes as a function of tillering potential. Sci Agric. 2009;66(1):28–39.

[33] Liao M, Fillery IR, Palta JA. Early vigorous growth is a major factor influencing nitrogen uptake in wheat. Funct Plant Biol. 2004;31(2):121–129. 32688884 10.1071/FP03060

[34] Aharon S, Peleg Z, Argaman E, Ben-David R, Lati RN. Image-based high-throughput phenotyping of cereals early vigor and weed-competitiveness traits. Remote Sens. 2020;12(29):3877.

[35] Whaley JM, Kirby EJ, Spink JH, Foulkes MJ, Sparkes DL. Frost damage to winter wheat in the UK: The effect of plant population density. Eur J Agron. 2004;21(1):105–115.

[36] Fossati D, Brabant C, Schori A. La Sensibilité Au Gel Des Variétés.ACW; 2012.

[37] McMaster GS, Wilhelm WW. Growing degree-days: One equation, two interpretations. Agric For Meteorol. 1997;87(4):291–300.

[38] Tschurr FR. *Experimentelle Untersuchungen über das Regenerationsverhalten bei alpinen Pflanzen*. Zürich: ETH Zürich; 1992.

[39] Jagadish KS, Way DA, Sharkey TD. Plant heat stress: Concepts directing future research. Plant Cell Environ. 2021;44:1992–2005. 33745205 10.1111/pce.14050

[40] Xin Z, Browse J. Cold comfort farm: The acclimation of plants to freezing temperatures. Plant Cell Environ. 2000;23:893–902.

[41] Zenkl R, Timofte R, Kirchgessner N, Roth L, Hund A, Van Gool L, Walter A, Aasen H. Outdoor plant segmentation with deep learning for high-throughput field phenotyping on a diverse wheat dataset. Front Plant Sci. 2021;12:774068. 35058948 10.3389/fpls.2021.774068 PMC8765702

[42] Lowe DG. Object recognition from local scale-invariant features. Paper presented at: Proceedings of the Seventh IEEE International Conference on Computer Vision; 1999 September 20–27; Kerkyra, Greece.

[43] Rublee E, Rabaud V, Konolige K, Bradski G. ORB: An efficient alternative to SIFT or SURF. Paper presented at: Proceedings of the 2011 International Conference on Computer Vision; 2011 November 6–13; Barcelona, Spain.

[44] Fischler MA, Bolles RC. Random sample consensus: A paradigm for model fitting with apphcatlons to image analysis and automated cartography. Graphics and Image Processing; 1981. p. 381–395.

[45] Virtanen P, Gommers R, Oliphant TE, Haberland M, Reddy T, Cournapeau D, Burovski E, Peterson P, Weckesser W, Bright J, et al. SciPy 1.0: Fundamental algorithms for scientific computing in python. Nat Methods. 2020;17:261–272. 32015543 10.1038/s41592-019-0686-2 PMC7056644

[46] Rife TW, Poland JA. Field book: An open-source application for field data collection on android. Crop Sci. 2014;54(4):1624–1627.

[47] R Core Team. R: A language and environment for statistical computing.Vienna: R Foundation for Statistical Computing; 2018.

[48] Varadhan R, Gilbert PD. BB: An R package for solving a large system of nonlinear equations and for optimizing a high-dimensional nonlinear objective function. J Stat Softw. 2009;32(4):1–26.

[49] Byrdt RH, Lut P, Nocedalt J, Zhu C. A limited memory algorithm for bound constrained optimization. SIAM J Sci Comput. 1995;16(5):1190–1208.

[50] Raffo MA, Sarup P, Andersen JR, Orabi J, Jahoor A, Jensen J. Integrating a growth degree-days based reaction norm methodology and multi-trait modeling for genomic prediction in wheat. Front Plant Sci. 2022;13:3138. 10.3389/fpls.2022.939448 PMC9481302 36119585

[51] Aslam MA, Ahmed M, Stöckle CO, Higgins SS, Hassan F, Hayat R. Can growing degree days and photoperiod predict spring wheat phenology? Front Environ Sci. 2017;5:57.

[52] Zou Y, Saddique Q, Dong W, Zhao Y, Zhang X, Liu J, Ding D, Feng H, Wendroth O, et al. Quantifying the compensatory effect of increased soil temperature under plastic film mulching on crop growing degree days in a wheat-maize rotation system. Field Crop Res. 2021;260: Article 107993.

[53] Wang E, Engel T. Simulation of phenological development of wheat crops. Agric Syst. 1998;58(1):1–24.

[54] Brown H, Huth N, Holzworth D. Crop model improvement in APSIM: Using wheat as a case study. Eur J Agron. 2018;100:141–150.

[55] Barnabás B, Jäger K, Fehér A. The effect of drought and heat stress on reproductive processes in cereals. Plant Cell Environ. 2008;31(1):11–38. 17971069 10.1111/j.1365-3040.2007.01727.x

[56] Shah F, Huang J, Cui K, Nie L, Shah T, Chen C, Wang K. Impact of high-temperature stress on rice plant and its traits related to tolerance. J Agric Sci. 2011;149(5):545–556.

[57] Fahad S, Hussain S, Saud S, Khan F, Hassan S, Amanullah, Nasim W, Arif M, Wang F, Huang J. Exogenously applied plant growth regulators affect heat-stressed Rice pollens. J Agron Crop Sci. 202(2):139–150.

[58] Millet EJ, Kruijer W, Coupel-Ledru A, Alvarez Prado S, Cabrera-Bosquet L, Lacube S, Charcosset A, Welcker C, Van Eeuwijk F, Tardieu F. Genomic prediction of maize yield across European environmental conditions. Nat Genet. 2019;51(6):952–956. 31110353 10.1038/s41588-019-0414-y

[59] Heslot N, Akdemir D, Sorrells ME, Jannink JL. Integrating environmental covariates and crop modeling into the genomic selection framework to predict genotype by environment interactions. Theor Appl Genet. 2014;127:463–480. 24264761 10.1007/s00122-013-2231-5

[60] Smith DT, Potgieter AB, Chapman SC. Scaling up high-throughput phenotyping for abiotic stress selection in the field. Theor Appl Genet. 2021;134:1845–1866. 34076731 10.1007/s00122-021-03864-5

[61] Chenu K, Porter JR, Martre P, Basso B, Chapman SC, Ewert F, Bindi M, Asseng S. Contribution of crop models to adaptation in wheat. Trends Plant Sci. 2017;22(6):472–490. 28389147 10.1016/j.tplants.2017.02.003

[62] Cooper MA, Messina CD, Podlich D, Totir LR, Baumgarten A, Hausmann NJ, Wright D, Graham G, Radu L, Totir B, et al. Predicting the future of plant breeding: Complementing empirical evaluation with genetic prediction. Crop Pasture Sci. 2014;65:311–336.

[63] Roth L, Fossati D, Krähenbühl P, Walter A, Hund A. Image-based phenomic prediction can provide valuable decision support in wheat breeding. bioRxiv. 2022. 10.1101/2022.09.07.506898. PMC10299972 37368140

[64] Tollhurst DJ, Mathews KL, Smith AB, Cullis BR. Genomic selection in multi-environment plant breeding trials using a factor analytic linear mixed model. J Anim Breed Genet. 2019;136:279–300. 31247682 10.1111/jbg.12404

[65] Tolhurst DJ, Chris Gaynor R, Gardunia B, Hickey JM, Gorjanc G. Genomic selection using random regressions on known and latent environmental covariates. Theor Appl Genet. 2022;135(10):3393–3415. 36066596 10.1007/s00122-022-04186-w PMC9519718

