# Supplementary material for: Frost Damage Index: The Antipode of Growing Degree Days
Source: Plant Phenomics. 2023 Oct 4;5:0104. doi: 10.34133/plantphenomics.0104 (PMC10550053; doi:10.34133/plantphenomics.0104)
Supplement: Supplementary 1 — Table S1. Spearman’s rank correlation coefficients between s and the visual scoring (VS) respectively of FIP measurements. Fig. S1. Overview of the 3 measurement years. Fig. S2. Visual ratings versus inverse ΔCC value for measurements in the year 2018 (days after sowing: 140) and 2019 (days after sowing: 212). Fig. S3. Prediction accuracy and RMSE over 0 to 10 d of lag time between FDI and daily interpolated ΔCC values. Fig. S4. Prediction accuracy and RMSE over 2 to 56 h of smoothing window of the temperature values. Fig. S5. Prediction accuracy using different Tbase temperatures. [file plantphenomics.0104.f1.pdf]

A Supplementary Materials

A.1 Supplementary table

- Correlations between measurements, visual scorings and optimized parameters: Table A.1

Table A.1: Spearman's rank correlation coefficients between s and the visual scoring (VS) respectively of FIP measurements. In 2018 negative ΔCC was taken from DAS 140, in 2019 from DAS 212. Pearson's correlation coefficients are shown between negative ΔCC and visual scoring of the years 2018 and 2019.

	s_{genotype}	- ΔCC 2018	- ΔCC 2019	VS 2018	VS 2019
All - ΔCC	0.897				
- ΔCC 2018		1			
- ΔCC 2019	0.52	0.522	1		
VS 2018	0.62	0.624	0.253	1	
VS 2019	0.48	0.49	0.321	0.392	1

A.2 Supplementary figures

- Overview of the three measurement years: Figure A.1
- Correlation between visual rating and inverse ΔCC measurements: Figure A.2
- Lag time determination: Figure A.3
- Smoothing factor determination: Figure A.4
- Evaluation of T_{base}: Figure A.5

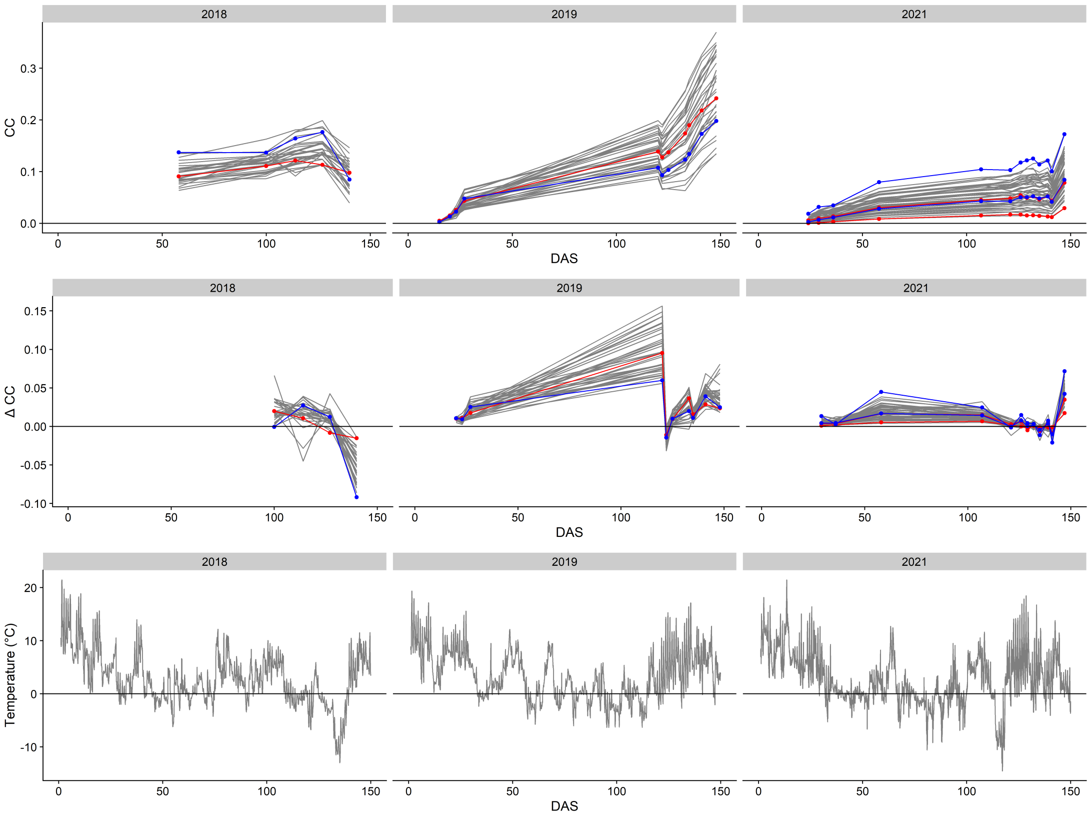

Figure A.1: Overview of the three measurement years. The first row shows the development of the CC over time for the different measurement years (columns). The second row shows the corresponding differences in CC between measurements (ΔCC), and the last row the temperatures over the measurement period. To illustrate the development of the two contrasting varieties from Figure 1, the variety CH Combin is represented in red and the variety Runal in blue.

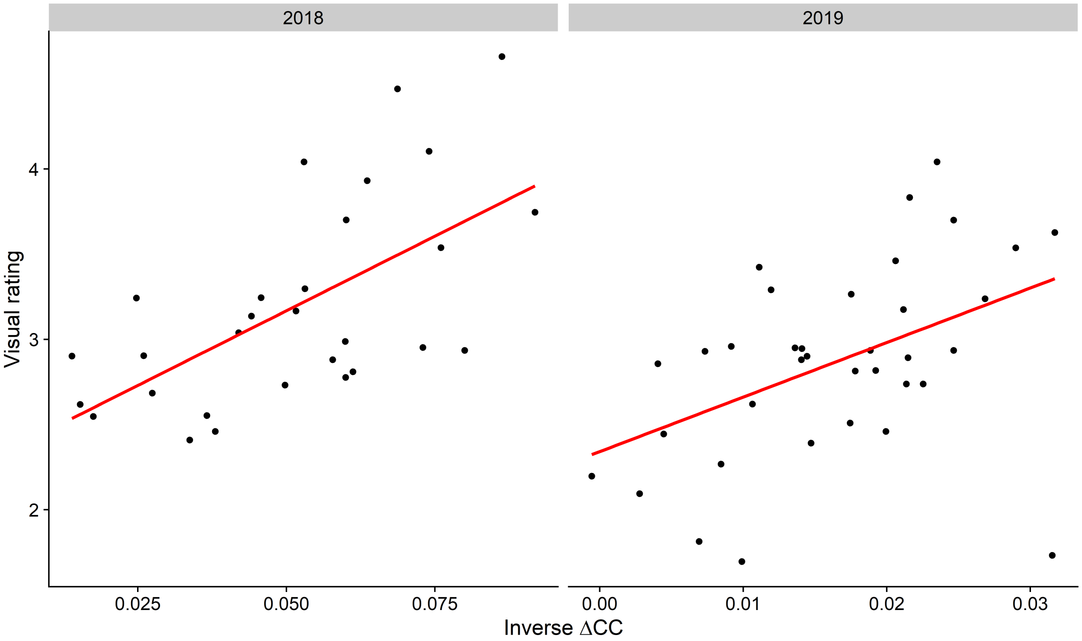

Figure A.2: Visual ratings versus inverse ΔCC value for measurements in the year 2018 (days after sowing: 140) and 2019 (days after sowing: 212). The red line shows the trend of the correlation.

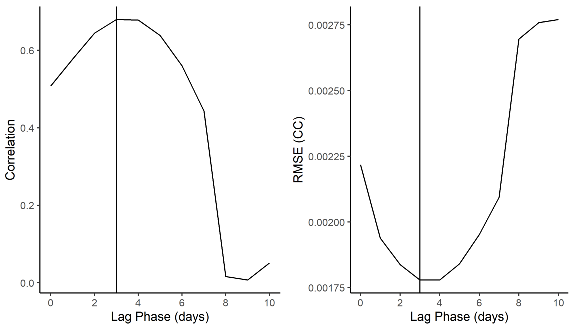

Figure A.3: Prediction accuracy and RMSE over 0 to 10 days of lag time between frost damage index (FDI) and daily interpolated ΔCC values. Highest prediction accuracy and lowest RMSE were found for 3 days (vertical lines).

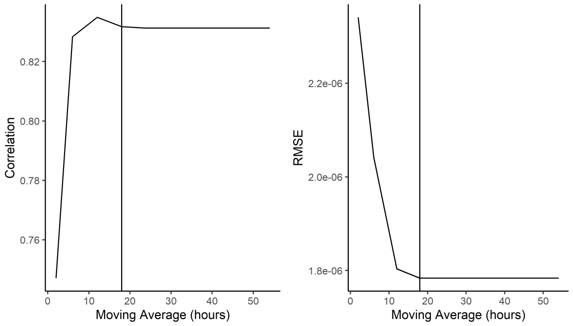

Figure A.4: Prediction accuracy and RMSE over 2 to 56 hours of smoothing window of the temperature values. The lowest RMSE was found for 18 hours, where the corresponding prediction accuracy is close to the maximum (vertical lines).

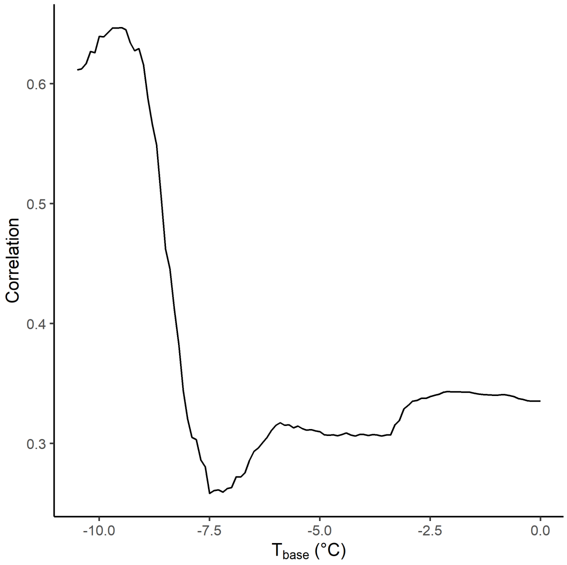

Figure A.5: Prediction accuracy using different T_{base} temperatures. The maximum correlation was achieved at -9.5 °C and rounded up to -9 °C as next integer.
